# Physical activity and the prevention, reduction, and treatment of alcohol and/or substance use across the lifespan (The PHASE review): protocol for a systematic review

**DOI:** 10.1186/s13643-018-0674-0

**Published:** 2018-01-22

**Authors:** Tom P. Thompson, Adrian H. Taylor, Amanda Wanner, Kerryn Husk, Yinghui Wei, Siobhan Creanor, Rebecca Kandiyali, Jo Neale, Julia Sinclair, Mona Nasser, Gary Wallace

**Affiliations:** 1 Plymouth University Peninsula Schools of Medicine and Dentistry, Plymouth Science Park, Derriford, Plymouth, PL6 8BX UK; 2 Centre for Mathematical Sciences, School of Computing, Electronics and Mathematics, Plymouth University, Drake Circus, Plymouth, PL4 8AA UK; 3 Bristol Medical School, Bristol University, Oakfield grove, Clifton, Bristol, BS8 2BN UK; 4 King’s College London, Institute of Psychiatry, Psychology and Neuroscience, Denmark Hill, London, SE5 8BB UK; 5 Faculty of Medicine, University of Southampton, 4-12 Terminus Terrace, Southampton, SO14 3DT UK; 6 Plymouth City Council, Public Dispensary, Catherine Street, Plymouth, PL1 2AA UK

**Keywords:** Physical activity, Exercise, Alcohol use, Substance use, Addiction, Prevention, Harm reduction, Treatment, Mixed method

## Abstract

**Background:**

Alcohol and substance use results in significant human and economic cost globally and is associated with economic costs of £21 billion and £15billion within the UK, respectively, and trends for use are not improving. Pharmacological interventions are well researched, but relapse rates across interventions for substance and alcohol use disorders are as high as 60–90%. Physical activity may offer an alternative or adjunct approach to reducing rates of alcohol and substance use that is associated with few adverse side effects, is easily accessible, and is potentially cost-effective. Through psychological, behavioural, and physiological mechanisms, physical activity may offer benefits in the prevention, reduction, and treatment of alcohol and substance use across the lifespan. Whilst physical activity is widely advocated as offering benefit, no systematic review exists of physical activity (in all forms) and its effects on all levels of alcohol and substance use across all ages to help inform policymakers, service providers, and commissioners.

**Methods:**

The objectives of this mixed methods systematic review are to describe and evaluate the quantitative and qualitative research obtained by a diverse search strategy on the impact of physical activity and its potential to:

1. Reduce the risk of progression to alcohol and/or substance use (PREVENTION)
2. Support individuals to reduce alcohol and/or substance use for harm reduction (REDUCTION), and
3. Promote abstinence and relapse prevention during and after treatment for an alcohol and/or substance use disorder (TREATMENT).

With the input of key stakeholders, we aim to assess how what we know can be translated into policy and practice. Quantitative, qualitative, service evaluations, and economic analyses will be brought together in a final narrative synthesis that will describe the potential benefits of physical activity for whom, in what conditions, and in what form.

**Discussion:**

This review will provide details of what is known about physical activity and the prevention, reduction, and treatment of alcohol and/or substance use. The synthesised findings will be disseminated to policymakers, service providers, and commissioners in the UK.

**Systematic review registration:**

PROSPERO number: CRD42017079322.

**Electronic supplementary material:**

The online version of this article (10.1186/s13643-018-0674-0) contains supplementary material, which is available to authorized users.

## Background

### Rationale

Alcohol and substance use is common: globally, 5.9% and 1% of deaths are attributable to alcohol and illicit drug use, respectively [1]. In the UK, alcohol use is attributed to more than one in five deaths of men aged 16–54 years old [2], and alcohol harms are associated with an economic annual cost of around £21 billion (£3.5billion in healthcare [3]). Illicit drug use in the UK has an economic cost of around £15 billion [4] (£488 million through healthcare [5]), with nearly one in ten adults aged 16–59 in England and Wales having used illicit drugs in the past year [6]. Worldwide, alcohol-attributable deaths increased from 3.8% in 2004 [1] to 5.9% in 2012 [7], and illicit drug use levels have failed to decline between 2005 and 2010 [8], with a slight increase in the UK in recent years [6].

### Scope for identifying new interventions

Pharmacological interventions for alcohol and substance use disorders have been well researched and reported on for the management of withdrawal, dependence, and relapse prevention. The Cochrane Drug and Alcohol Group has published 11 and 30 reviews of pharmacological interventions for alcohol and substance use, respectively, whilst psychosocial interventions (e.g. brief interventions and motivational interviewing) are less well reported, with six and eight published reviews, respectively. Preventive interventions only have five reviews for alcohol use, and three reviews for substance use [9]. Due to the heterogeneity of the types of drugs used and style of intervention, it is hard to summarise meaningfully the available data of existing interventions. However, with relapse rates as high as 60% 1 year after treatment for substance use disorders (SUD) [10–12] and 60–90% for alcohol use disorders (AUD) [13–16] and drug substitution therapies being associated with innate complications [17–20], there is a need for evidence for new treatments and preventive interventions to help address the growing burden of alcohol and/or substance use.

Physical activity (PA; defined as any bodily movement produced by skeletal muscles that requires energy expenditure, inclusive of organised sport [21]) and health-oriented exercise interventions could impact on the prevention, reduction, and treatment of alcohol and/or substance use and have the potential to be cost-effective, flexible, accessible, acceptable across the range of levels of use and have a lower risk of adverse events compared to pharmacological treatment [22]. In 2001 (with updates in 2005 and 2008), AT (with co-researchers) reviewed and reported the effects of exercise on smoking from eight randomised controlled trials (RCTs) as part of a Cochrane Review [23]. This evidence contributed to a 2008 report to the US Surgeon General on smoking cessation which highlighted the value of exercise as an option to support smoking cessation [24], and in the UK, many NHS Stop Smoking Services now advocate exercise [25]. In 2014, an update to the Cochrane Review revealed there were 20 RCTs of exercise and smoking cessation, suggesting a rapid growth of interest in the topic [23]. A first systematic review is now needed of physical activity interventions for the prevention, reduction, and treatment of alcohol and/or substance use that also includes a comprehensive search of grey literature and service evaluations to generate practical implications for practice and policy.

### Evidence for the role of PA for preventing alcohol and/or substance use

Prospective studies indicate that sports participation in adolescents and young people is associated with an increase in alcohol use but decrease in illicit drug use [26]. However, such studies may fail to eliminate confounding factors (e.g., specific sports may attract those more predisposed to engaging in ‘risky’ behaviours).

In contrast, a rigorous study in Finland tracked 1870 twin pairs from 16 to 27 years of age and concluded that low levels of physical activity increased the risk of both alcohol and illicit drug use [27]. This further demonstrates the need for a robust, systematic review assessing the role of physical activity (not just participation in sports) on progression to alcohol and/or substance use disorders.

### Evidence for the effects of PA interventions for harm reduction and treatment of alcohol and/or substance use

There is increasing interest in the role of physical activity as a treatment and reduction strategy for alcohol and/or substance use. In 2011, the US National Institute on Drug Abuse (NIDA) invested $4.3 million [28] on a programme of work including high-quality RCTs such as STRIDE [29] which is investigating stimulant use reduction using exercise. A recent systematic review by Wang et al. [30] was limited by an incomplete search strategy (e.g., not CINAHL) and thus omitted key papers. They identified three studies with a focus on alcohol, five with a focus on illicit drug use, four on multiple drug use, and 11 on smoking. The data from each of these studies were pooled using meta-analysis, despite considerable apparent heterogeneity across interventions and outcomes. Harm-reduction studies were not considered. In another systematic review, Zschucke and colleagues [31] found nine studies reporting the effects of PA on AUD and eight on SUD, but again, that review did not include key search engines and did not consider grey literature that may be most informative for the UK context. Both these reviews focussed on AUD and SUD and did not consider the broader spectrum of use that may not meet the classification of a disorder, e.g. recreational use that is still associated with risk of harm. A rigorous review of the evidence encompassing all aspects of alcohol and substance use is still needed.

### Plausible mechanisms for the effects of exercise on the use of any addictive substance

Physical activity may affect alcohol and/or substance use through various psychological mechanisms, such as an acute reduction in cravings and urges, an increase in positive affect, and a chronic improvement in co-morbid depression and anxiety which may moderate outcomes related to alcohol and substance use [32]. From the behavioural perspective, exercise involvement may help avoidance of cues which trigger cravings and relapse, and provide exposure to new environments, which provide diversionary safe and immediately rewarding experiences [32]. Participation in meaningful structured activities are a key part of overcoming AUD and SUD, and some physical activities may offer the chance for identity transformation through exposure to meaningful routine activities, informal social controls, and promoted personal agency [33]. From the physiological perspective, there is evidence from animal studies to suggest that neurobiological changes associated with exercise [32, 34, 35] help to explain the consistent evidence that exercise acutely reduces consumption of cocaine, morphine, nicotine, and alcohol [34, 36–39].

Finally, recent studies indicate that physical activity interventions can be acceptable for those with AUD and SUD [40–42], but no review exists of this published and grey literature to help inform the design of the most feasible and acceptable interventions across the spectrum of levels of use.

PA may influence alcohol and/or substance use in similar ways and through common mechanisms and therefore form the focus of this review. However, due to the different way in which alcohol and substance use are viewed, approached, and treated within the UK, they will be considered separately within this review and not combined in any analyses in order to ensure the most pertinent findings for policy and practice.

### Impact of stakeholder engagement

Stakeholder engagement has many benefits and can contribute towards the development of systematic reviews. Stakeholders can be funders, service users, healthcare professionals, or charities, i.e. anyone who will implement interventions based on the findings of the reviews. A research white paper looking at the benefits of stakeholder engagement in systematic reviews was published by Cottrell and colleagues in 2014 [43]. They reviewed papers and suggested that the benefits of stakeholder engagement included identifying and prioritising potential research topics, helping to recruit participants, and providing useful feedback on the systematic review protocol. Other benefits included helping the researchers to understand the perspective of the service users/participants and ensuring the accessibility of the results with wider dissemination. Most studies reviewed were in the UK, and they suggested that the scope for the reviews was refined due to stakeholder engagement and that generally the overall quality of the review was improved. Given the broad focus of this review across several sectors and service providers in what is an under-researched area, involving stakeholders will maximise the applicability and impact of the findings.

As part of this process, our original questions were based on concerns highlighted by the Plymouth City Council Public Health team and the Plymouth NHS Hospital Trust due to the high national prevalence of alcohol and/or substance use and resulting hospital admissions in the area. We worked with a local third sector organisation which provides day support for persons in the community affected by the use of drugs and/or alcohol, as well as an education service as an alternative to pupil referral units in Plymouth. Stakeholder groups within this service (three service providers and eight service users) supported the focus on our three key research questions about prevention, harm reduction, treatment and relapse prevention, and highlighted the importance of PA through their own narratives, independent of, and in addition to, standard treatment. Further engagement with co-applicants Gary Wallace (Senior Specialist Drugs and Alcohol Team Manager in the Plymouth Public Health), Julia Sinclair (Honorary Consultant in Alcohol Liaison and Wessex Alcohol (AHSN) Lead), Joanne Neale (lead for PPI addiction research group at KCL), and local third sector leaders refined the scope and methods for the review. In addition, co-applicants Adrian Taylor and Joanne Neale have previously conducted and published qualitative research involving people with SUD which highlighted the need to further develop appropriate interventions [25, 42]. The scope and methods for this review have been strongly influenced by both service provider and user perspectives throughout the development of the application and this protocol.

### Aim and objectives

Our overarching aim is to describe and evaluate the quantitative and qualitative research on the impact of physical activity on the prevention, reduction, and treatment of alcohol and/or substance use across the lifespan.

Physical activity interventions (including those involving sport, exercise, or general lifestyle physical activity) may have the potential to impact three domains of alcohol and/or substance use:

1. Reduce the risk of progression to alcohol and/or substance use (PREVENTION);
2. Support individuals to reduce alcohol and/or substance use for harm reduction (REDUCTION), and
3. Promote abstinence and relapse prevention during and/or after treatment of AUD and SUD (TREATMENT).

We aim to describe and evaluate the available quantitative and qualitative research for each of these scenarios and seek to assess how what we know can best be translated into policy and practice with the input of key stakeholders, presenting where possible any cost-effectiveness data.

This will be achieved by the following objectives:

- To quantify and describe quantitative data relating to the impact of physical activity on alcohol and/or substance use outcomes (completing meta-analyses where possible);
- To analyse and describe qualitative data relating to the acceptability, feasibility, mechanisms, mediators, and moderators of physical activity in relation to alcohol and/or substance use (completing meta-syntheses where possible);
- To describe and analyse service evaluations which may not meet peer-reviewed quantitative or qualitative inclusion criteria relating to the implementation and impact of physical activity interventions relating to alcohol and/or substance use;
- To quantify and describe potential cost-effectiveness data relating to physical activity and its impact on alcohol and/or substance use;
- To produce practical recommendations about what is known about what works for who, when, where, and how through a narrative synthesis informed by stakeholder input.

Each of these objectives will address the three domains of prevention, reduction, and treatment, separately, but some crossover will be expected.

## Methods

This protocol has been prepared using the Preferred Reporting Items for Systematic Reviews and Meta-Analyses protocols (PRISMA-P) guidelines [44] (see Additional file 1).

### Eligibility criteria

We will not limit our searches by country; however, we will only include papers published in English. Whilst we recognise there is a potential for bias to be introduced because of limiting the searches to English, the direction and degree of such bias are unknown. As outlined in the Cochrane Handbook for Systematic Reviews of Interventions [45], there is conflicting evidence about the potential bias introduced by an English language limit: Juni [46] reported that non-English trials were more likely to report significant results, whilst Moher [47] reported no significant difference in meta-analyses which excluded trials in languages other than English. Studies will be restricted from 1978 to the present: 1978 was chosen as a cutoff point based on a frequency analysis on a subsample of relevant literature.

### Types of studies

We will include (a) quantitative studies (RCTs, quasi-RCTs, non-randomised controlled trials, controlled before and after studies, prospective or retrospective cohort studies that include a control group, historically controlled trials, nested case-control studies, case-control studies, and before-and-after comparisons); (b) qualitative investigations (of any recognised qualitative methodology); (c) local service evaluations; and (d) economic evaluations (full and partial).

### Type of setting

We will not limit the setting or country in which interventions are delivered (although this will be impacted considerably by the English language restriction), and this variation will be considered in the narrative synthesis. Studies may include inpatient and outpatient programmes, public health interventions, and community-based interventions. We will not place any limitations on who delivers the intervention and in what format.

### Participants/population

No limit on participants will be applied; all adults and children will be considered. We expect most studies to include adolescents at risk of alcohol and substance use (prevention), adults in acute rehabilitation for SUD/AUD and post-acute rehabilitation for SUD/AUD (relapse prevention and supporting abstinence), and any other adults receiving support or intervention for reducing alcohol and substance use (reduction). We will record and consider these diversities in the synthesis of results. We expect certain populations to be of particular significance in the research (e.g. people who are homeless, have mental health problems, or belong to groups experiencing complex needs or disadvantages), where alcohol and substance use may not be the primary outcome and physical activity may be part of a more complex intervention. Where this type of study is identified, it will be assessed for relevance on a case-by-case basis, discussed within the research team, and included if it contains viable data that can be included within the review’s defined primary outcomes.

### Intervention(s) and comparator(s)

We will include any studies evaluating and comparing interventions that include a physical activity promotion element either explicitly targeting a reduction in alcohol and substance use or implicitly resulting in a reduction in alcohol and substance use. This could be within one of the three domains of prevention, reduction, or treatment. The comparator could be no intervention, treatment as usual (e.g. pharmacotherapy and psychological therapies), or alternative physical activity interventions (e.g. running vs walking).

The scope of this review is to include research on alcohol and substance use in its broadest sense. We plan to include data on alcohol and substance use which may not be considered a ‘disorder’ which reflects levels and prevalence of use, as well as including research on AUD and SUD as classified in the diagnostic and statistical manual of mental health disorders, fifth edition (DSM-V) [48].

### Outcomes

The primary outcomes are mapped against the four planned analyses by the three domains of PA and its possible impact on alcohol and/or substance use in Table 1.

**Table 1:** Outcomes tabled by population, intervention, control, and outcome against the three domains of physical activity and its possible impact on alcohol and substance use

	(1) Prevention	(2) Reduction	Treatment (and relapse prevention)
Population	Adolescents, at risk groups	General population who use alcohol or substances but not receiving acute or long-term care for a diagnosed AUD and/or SUD, at risk groups	Those receiving/have received acute or long-term care for a diagnosed AUD and/or SUD
Intervention	Sport and physical activity-based programmes (schools, community, public health interventions)	Public health level initiatives, targeted community- and healthcare-based interventions	Adjunct PA interventions, prescribed and supported PA interventions, motivational interventions
Control	Other non-PA control, usual care, or no intervention	Other non-PA control, usual care, or no intervention	Standalone usual care, non-PA control
Outcome			
(a) Quantitative^a^	Levels of subsequent use of alcohol and/or substances, prevalence rates	% reduction in alcohol and/or substance use, prevalence	Abstinence rates, % days abstinent, % reduction in alcohol and/or substance use, relapse rates
(b) Qualitative	Acceptability, feasibility, barriers and facilitators of PA	Acceptability, feasibility, barriers and facilitators of PA, perceived utility of PA	Acceptability, feasibility, barriers and facilitators of PA, perceived utility of PA
(c) Service Evaluations	Mixture of above (a) and (b) outcomes, implementation issues	Mixture of above (a) and (b) outcomes, implementation issues	Mixture of above (a) and (b) outcomes, implementation issues
(d) Economic	Costs, cost-effectiveness, net benefit, resource use and cost, economic measures of benefit (e.g. Quality-adjusted-life-years (QALYs), and willingness to pay (WTP)), summary economic measures (e.g. incremental cost-effectiveness ratios ICERs)	Costs, cost-effectiveness, net benefit, resource use and cost, economic measures of benefit (e.g. Quality-adjusted-life-years (QALYs), and willingness to pay (WTP)), summary economic measures (e.g. incremental cost-effectiveness ratios ICERs)	Costs, cost-effectiveness, net benefit, resource use and cost, economic measures of benefit (e.g. Quality-adjusted-life-years (QALYs), and willingness to pay (WTP)), summary economic measures (e.g. incremental cost-effectiveness ratios ICERs)

^a^Outcomes listed are indicative and not exhaustive

#### Secondary outcomes

Secondary outcomes will be collected during the data extraction phase in addition to the primary outcomes above where present. These include:

- Physical activity levels/fitness;
- Biomedical outcomes (e.g. liver function, hepatitis C status);
- Mental health and wellbeing;
- Adverse events.

We will also extract data referring to the identification of the underlying psychological theory informing interventions; intervention structure and content; information relating to how an intervention may work including challenges, barriers, and facilitators of behaviour change (process evaluations); the mechanisms of change (mediators and moderators), acceptability, and feasibility data; and any evidence of a dose-response relationship.

### Information sources

We will develop and test a highly sensitive search strategy of published and grey literature using background scoping searches, previously identified relevant research, and in consultation with subject experts and public and patient involvement. The strategy will include searches of the following sources:

#### Database searching

- MEDLINE (Ovid)
- MEDLINE (PubMed)
- Embase (Ovid)
- PsycINFO (Ovid)
- Cochrane Library (Wiley) (including Cochrane Database of Systematic Reviews, Cochrane Central Register of Controlled Trials, Database of Abstracts of Reviews of Effects, Health Technology Assessment Database, and NHS Economic Evaluation Database)
- International Bibliography of the Social Sciences (ProQuest)
- Web of Science Core Collection
- CINAHL (EBSCO)
- AMED (EBSCO)
- Social Policy and Practice (Ovid)
- Applied Social Sciences Index and Abstracts (ProQuest)
- SocINDEX (Ebsco)
- SportDiscus (Ebsco)

#### Supplementary database searches

- Google and Google Scholar
- Open Grey
- ProQuest Dissertations & Theses
- British Library EThOS
- Scottish Addiction Studies online library
- HRB National Drugs Library
- NIDA International Drug Abuse Research Abstract Database
- Tufts CEA Registry
- Database of promoting health effectiveness reviews (DoPHER)
- NHS Evidence (NICE)
- Big Lottery Fund Database

### Search strategy

An Information Specialist (AW) will design and conduct the search strategy with expert consultation. The strategy will be translated for use in each database stated above, and a modified keyword-only strategy will be used for grey literature searching. The search strategy will be designed to encompass the three aims of the review (i.e. prevention, reduction, and treatment).

See Appendix 1 for a sample search strategy.

### Searching other resources

Extensive grey literature searching will be conducted to ensure maximum coverage of the subject area. The grey literature strategy will encompass focused searches in Google, several specialised databases, and consultation of subject experts for recommendations. This process will generate grey literature publications as well as relevant websites of local and national organisations in the UK, which will be hand-searched for additional citations. We will also conduct backwards and forwards citation chaining of all included studies to identify further relevant articles, as well as directly contact-known experts in the field and the lead authors of key publications for knowledge of any other relevant work. We will include PhD theses, but exclude MSc theses.

All grey literature websites and search engines will be searched with targeted keywords and phrases generated from our original search strategy. The first 100 hits of each search will be screened by title and abstract. If a high proportion of the first 100 hits (≥ 10%) can potentially be included, then a further 100 hits will be searched continuing until the next 100 hits contain ≤ 10% of potentially includable hits. If the initial search produces fewer than 100 hits, then all hits will be searched.

See Appendix 2 for sample grey literature search strategy.

### Study records

#### Data management

Exported citations from traditional databases will be entered and de-duplicated into EndNote X8 (Clarivate Analytics). Grey literature results will be manually entered or, where available, captured through a browser-based citation management plug-in (such as Zotero [https://www.zotero.org/]) then imported into EndNote. Using a structured and piloted data extraction form, we will extract relevant outcome data, study characteristics, and participant characteristics from each included paper. Data will be extracted by one reviewer and checked by another.

### Selection process

#### Indexed and academic databases

Two waves of study selection will be undertaken. Titles and abstracts will be screened by two reviewers independently and disagreements resolved by discussion or, where necessary, a third reviewer (title and abstract screening will be conducted using Rayyan software (QCRI; Doha, Qatar; https://rayyan.qcri.org/)). Two initial subsets of 500 results will be screened by two reviewers and inclusion and exclusion discrepancies discussed following each in order to ensure good agreement between reviewers. Following this, a set of 1000 will be completed and discussed before the remaining results being screened independently by two reviewers. This will help ensure reliable and consistent screening. Full texts will be obtained for studies appearing to meet the criteria above and screened by two reviewers (each paper reviewed by one member of the team and checked by another). Disagreements are resolved through discussion and a third reviewer (AT). RK will be consulted in relation to uncertainty over economic evaluations arising from the two independent reviewers.

The same process will apply for grey literature searching.

### Appraisal of studies (quality and bias)

We will evaluate risk of bias at the level of outcomes. Randomised controlled trials will be assessed for quality and risk of bias using the Cochrane Risk of Bias Tool [49], and non-randomised studies will be assessed using the ROBINS-I [50]. Any economic evaluations will be assessed for study quality using the Consolidated Health Economics Evaluation Reporting Standards (CHEERS) [51] checklist. Qualitative studies will be assessed for quality using a ten-item checklist for qualitative studies developed and published by the Critical Skills Appraisal Programme (CASP) [52] which focuses on rigour, credibility, and relevance without being overly restrictive. CASP will be used to appraise studies but not to exclude any studies.

### Data synthesis

Data synthesis will be adapted from the multilevel approach as suggested in the Cochrane Handbook of Systematic Reviews: Quantitative and qualitative evidence will be reviewed separately and then combined into an overall narrative synthesis. A narrative synthesis of service evaluations and economic data will be integrated into the main synthesis to aid in contextualising the results in terms of implementation.

### Quantitative studies (analysis A)

Where data allow (e.g. data on the same outcome from at least two studies of similar design, intervention, and population), we will conduct a meta-analysis to estimate the overall effect and consistency of the intervention effect across studies. As the population and setting of studies are likely to be different, we will use a random-effects model to obtain the summary result as an estimate of the average intervention effect rather than the common effect estimated from a fixed effects model [53]. Where possible, we will create and examine funnel plots for the association between study size and estimated effect size, which could be due to publication bias. Where possible, we will explore the extent to which the intervention characteristics, study setting (country, socioeconomic status, healthcare system), and participant characteristics moderate the effect of interventions, through conducting meta-regressions or subgroup analyses.

We will not combine data from non-randomised trials which used different study designs, or data from randomised trials and non-randomised trials, in a meta-analysis, as the estimated intervention effects from different study designs can be influenced by different sources of bias and/or increased heterogeneity [45]. In those cases, where suitable numerical data are not available for pooling, or if pooling is considered inappropriate, we will use other approaches to provide a systematic summary of the studies, including tabulation, transformation of data into common rubric (e.g. days abstinent), groupings and clusters (e.g. different population to assess influence of country, age, socioeconomic status, type/intensity of intervention, setting), and textual descriptions including a detailed narrative synthesis [54].

### Qualitative studies (analysis B)

The qualitative synthesis aims to describe qualitative data relating to the acceptability, feasibility, mechanisms, mediators, and moderators of physical activity in relation to alcohol and/or substance use. Data on the development, design, methods, and the populations involved will be extracted from qualitative studies using a bespoke data extraction form. The complete “findings” or “results” sections of the qualitative study reports will be exported into NVivo 10 (QSR International Pty Ltd.). Each section will then be read and re-read by two reviewers, in conjunction with the data extraction form, to enable the reviewers to familiarise themselves with the study findings in the context of the study population, setting, and methods. Adopting a thematic analysis approach, reviewers will code and identify emergent themes and concepts independently (extracting associated quotes). The reviewers will come together to consolidate the findings into one summary of overarching themes. Associated quotes will be presented to support the identified themes. The review team will then draw out implications of the themes for policy and practice.

### Service evaluations (analysis C)

Service evaluations will be considered separately from the academic literature, and through a thematic synthesis approach will be summarised to help understand contextual and implementation issues surrounding the delivery of PA for alcohol and/or substance use. It will also be used, where possible, to contextualise data from the academic literature within the UK context to aid with the final narrative synthesis.

### Economic evaluations (analysis D)

The review of resource use, costs, relative effectiveness and cost-effectiveness will include a descriptive summary of the (economic) study questions, methods, and results, culminating in a narrative synthesis. Since the purpose of our review is to provide clear and concise information on the existing economic evidence base, we will also consider partial economic evaluations. These may include cost comparisons, as well as studies with an exclusive focus on relative benefits, i.e. studies that discuss willingness to pay or preference-based outcome measures. Summary tables will not be limited to description of economic outcomes alone and will include all relevant information integral to the economic study. We will extract detail on analytic methods, study perspective, price year, country, currency, and time horizon with further extraction fields informed by section headings within the CHEERS [51] checklist. Since we do not anticipate a substantial amount of economic literature, our methods may focus on translating findings from the review for the purposes of dissemination and stakeholder input. All types of comparative economic study design, including decision-analytic modelling approaches, will be included.

### Narrative synthesis

The analysis of the quantitative (analysis A), qualitative (analysis B), service evaluation (analysis C), and economic (analysis D) data will be integrated to develop a narrative synthesis. This will be summarised for dissemination to PPI groups and key stakeholders and used as a basis for generating critical input to help understand the implications of the findings for different groups.

### Measures of intervention effects (quantitative data)

#### Dichotomous data

We will present dichotomous data as risk ratios with their associated 95% confidence intervals (CI).

#### Continuous data

For continuous data, we will calculate the mean differences (MD) for outcomes measured by the same scale or the standardised mean differences (SMD) for outcomes measured by different scales and present both with a 95% CI.

#### Outcomes at multiple time points

If outcomes were collected at multiple time points, we will attempt to present a summary effect over all time points. If this is not possible, we will choose one time point that is the most appropriate one and report the corresponding summary effect at that time point.

### Unit of analysis issues

Cluster randomised trials are susceptible to unit-of-analysis errors if the analysis was performed at the level of the individual without accounting for the clustering in the data. If the clustering effect has been accounted for in the analysis, the estimated intervention effect will be obtained from the reported summary data. If the clustering effect has not been accounted for, we will conduct an approximate analysis using the intra-cluster correlation coefficient (ICC), as suggested in the current guidelines [45]. If the ICC is available in the study reports or can be obtained from similar studies, we will use the available ICC to calculate the inflated standard error or effective sample size to account for the clustering effect. If a relevant ICC is not available, we will report the estimated intervention effect as presented but report the issue of unit of analysis error.

### Dealing with missing data

If a study did not provide the summary data of the intervention effects, we will contact the study authors on one occasion to request these data. Where individual-level data are missing due to participant dropout, we will conduct available case analyses and record any issues of missing data in the ‘Risk of bias’ table. If standard error is available but standard deviations are not reported in a study, we will estimate the standard deviation from the reported standard error and the sample size. We will calculate the effect estimate and its standard deviation if these are not reported, but the 95% CI is reported.

### Assessment of statistical heterogeneity

We will assess statistical heterogeneity by inspection of forest plots, and by formal statistical tests of homogeneity (Chi-squared), measures of inconsistency (*I*^2^) [55], and between-study variance (tau^2^). We interpret the level of heterogeneity as follows: the heterogeneity is not important if *I*^2^ is lower than 40%; there is moderate heterogeneity if *I*^2^ is between 30 and 50%; and there is substantial or considerable heterogeneity if *I*^2^ is greater than 50% [45]. If substantial heterogeneity is identified among studies, we will explore the potential causes of heterogeneity by conducting subgroup analyses or meta-regression where possible.

### Assessment of reporting biases

We will examine the possibility of publication bias and other small study effects using funnel plots of the intervention effect estimates against the inverse of their standard errors and test funnel plot asymmetry using Egger’s method [56] when there is a sufficient number of studies. This is considered at least ten, as a smaller number would leave the power of the test too low to distinguish chance from real symmetry.

### Sensitivity analysis

We will conduct a sensitivity analysis based on quality indicators thought to be significant by the review team. Studies thought to be at high risk of bias due to specific quality indicators (e.g. lack of randomisation) will be removed to ascertain their effect on the estimated overall effect. We also intend to conduct a sensitivity analysis using fixed-effect models.

### Confidence in cumulative evidence

The quality of evidence will be assessed using domains of the Grading of Recommendations, Assessment, Development, and Evaluation (GRADE) [57] guidelines. Strength of evidence will be judged as ‘high’ (further research is very unlikely to change confidence in our findings), ‘moderate’ (further research is likely to have an important impact on our findings), ‘low’ (further research is likely to have an important impact and change our findings), ‘very low’ (further research is needed to draw any conclusions).

### External validity/generalisability

We will explore generalisability at the study level and on the level of the aggregated evidence.

We will collect the following data from each study: details of the intervention, fidelity of the intervention and adherence to it (this includes who delivers the intervention), rationale supporting the choice of outcome measures, and setting in which the study was conducted (country, socioeconomic status of the setting, healthcare system). Depending on the type of available data, we will analyse them using subgroup analyses, meta-regression, or contextualise it through narrative synthesis.

### Advisory groups

We will establish advisory groups: consisting of academics, service providers, and service users. These groups will offer varying perspectives on pertinent issues arising and will provide input into different aspects of the review.

The academic group will be asked to provide a critical voice on the interpretation of findings, to ensure no aspect has been overlooked, and to aid in establishing networks for finding data and disseminating results.

The service provider group will offer insight into the UK context in which alcohol and substance use exists, what the commissioning landscape looks like, current and future reach and structure of services and interventions, contextualise findings to the current UK climate, and to aid in dissemination and implementation of results.

The service user group will offer input into what outcomes we should be searching for, approaches to promote physical activity (i.e. sport, exercise, and daily activity) would be most acceptable to people; and in what other ways could support services (who, where, when) be set up to maximise the reach and effectiveness for promoting physical activity for the prevention, reduction, and treatment of alcohol and substance use.

To maximise the impact of stakeholder, public, and patient involvement, we will develop a user-friendly synthesis of the findings and nature of interventions and their apparent strengths and weaknesses working with the advisory groups. Once a user-friendly synthesis has been generated, it will be disseminated to key stakeholder groups and individuals and their feedback and input will be used to gain further insights into what the evidence suggests and where any gaps may exist. Specifically, people will represent different stages of addiction, including those who are occasional users at risk of progressing to regular users of alcohol and/or substance use, non-treatment seekers who wish to minimise harm, and those currently receiving treatment or who are in recovery. This synthesis will then be disseminated to key stakeholder groups and individuals and used as the basis for several group and individual meetings to gain further input into what the evidence suggests and where any gaps may exist.

### Dissemination and intended publications

Upon completion of the review, we will develop a summary of key findings of the review of literature, a summary of the PPI assessment of the findings, and issues associated with service development and delivery highlighted by policy makers and service managers. We will present the findings at relevant academic conferences and a website that will be established to summarise the findings and implications, with links to access a final report. We intend also to organise a one-day conference to which key stakeholders, advisory board members, and any interested party will be invited. In addition to a final report, we anticipate submitting articles for publication in peer-reviewed open access journals.

We will also disseminate the findings by phone or Skype to Directors of Public Health (or leads for alcohol and substance misuse) and managers of organisations across the UK who do or could involve physically active interventions to gain a further insight into the issues associated with securing the necessary resources.

## Discussion

The scope and methods for this review have been strongly influenced by both service provider and user perspectives throughout the development of the application and this protocol. The review will generate important and timely information to inform the provision of services for alcohol and substance use. Through the continued engagement with stakeholders, the information produced will have relevance across a variety of settings in addressing the prevention, reduction, and treatment of alcohol and/or substance use throughout the UK. A wide variety of dissemination plans will ensure the information is accessed by the most relevant services, as well as aiding to direct future research efforts.

The size and scope of the review, whilst challenging, will ensure that the information brought together in this review will be as encompassing as possible and will provide all the necessary information about what we know about what works, for who, when, where, and how in an accessible and appropriate way. Information generated from this review will have the potential to directly impact on provision in several domains and address (where possible) issues of acceptability, feasibility, implementation, and cost-effectiveness.

## Appendix 1

### Sample search strategy

#### Databases to be searched

To be conducted by AW.

- MEDLINE (Ovid)
- MEDLINE (PubMed) Note: supplementary search only
- Embase (Ovid)
- PsycINFO (Ovid)
- Cochrane Library (Wiley)
- International Bibliography of the Social Sciences (ProQuest)
- Web of Science
- CINAHL (Ebsco)
- AMED (EBSCO)
- Social Policy and Practice (Ovid)
- Applied Social Sciences Index and Abstracts (ProQuest)
- ProQuest Dissertations & Theses (ProQuest)
- SocIndex (Ebsco)
- SportDiscus (Ebsco)

#### Sample database search to be translated into the databases above

Database: MEDLINE

Host: Ovid

Data parameters: Ovid MEDLINE(R) Epub Ahead of Print, In-Process & Other Non-Indexed Citations, Ovid MEDLINE(R) Daily and Ovid MEDLINE(R) 1946 to Present

Date searches: 2-jun-17

Searcher: AW

Hits: 12,860

Strategy:

**Table:**

#	Searches	Results
1	exp exercise/	157,603
2	exp exercise therapy/	41,209
3	exp exercise movement techniques/	6736
4	sedentary lifestyle/	5870
5	exercis*.ti,ab,kw.	255,603
6	Fitness.ti,ab,kw.	57,641
7	sport*.ti,ab,kw.	61,127
8	isometric.ti,ab,kw.	29,699
9	yoga.ti,ab,kw.	3404
10	tai chi.ti,ab,kw.	1308
11	qigong.ti,ab,kw.	519
12	walk*.ti,ab,kw.	96,513
13	Jog*.ti,ab,kw.	2103
14	(weight lifting or weightlifting).ti,ab,kw.	1195
15	sedentary.ti,ab,kw.	24,682
16	pedometer*.ti,ab,kw.	2223
17	aerobic*.ti,ab,kw.	74,000
18	(physical adj1 (train* or program* or activit* or inactivity or fitness or conditioning)).ti,ab,kw.	100,132
19	((resistance or strength or endurance or weight) adj2 train*).ti,ab,kw.	17,368
20	or/1-19 [ physical activity terms ]	630,395
21	Substance-Related Disorders/	88,741
22	Alcohol-Related Disorders/	4588
23	Alcohol-Induced Disorders/	251
24	Alcoholic Intoxication/	12,082
25	Alcoholism/	72,144
26	Binge Drinking/	1060
27	Amphetamine-Related Disorders/	2676
28	Cocaine-Related Disorders/	7413
29	Drug Overdose/	9375
30	Inhalant Abuse/	176
31	Marijuana Abuse/	5487
32	Opioid-Related Disorders/	10,773
33	Heroin Dependence/	8695
34	Morphine Dependence/	3356
35	Phencyclidine Abuse/	238
36	Psychoses, Substance-Induced/	5159
37	Substance Abuse, Intravenous/	13,950
38	Substance Withdrawal Syndrome/	20,489
39	Alcohol Withdrawal Delirium/	1900
40	Alcohol Withdrawal Seizures/	244
41	alcoholics/	788
42	drug users/	2194
43	underage drinking/	293
44	Alcohol Drinking in College/	200
45	designer drugs/	1294
46	exp street drugs/	10,924
47	prescription drug misuse/	1072
48	Alcohol Abstinence/	350
49	Alcohol Drinking/	60,264
50	((alcohol or ethanol or drug* or inhal* or inject* or substance) adj2 (abstinence or abstain or abuse or craving* or dependenc* or illegal or illicit or misuse or overdos* or prevent* or recovery or recreational or use* or withdrawal)).ti,ab,kw.	243,666
51	(prescription adj2 (abuse or craving* or dependenc* or illegal or illicit or misuse or nonmedical or non-medical or overdos* or recreational or withdrawal)).ti,ab,kw.	1963
52	(hard* adj1 drug*).ti,ab,kw.	550
53	(withdrawal adj2 (symptom* or syndrome*)).ti,ab,kw.	8682
54	hazardous drinking.ti,ab,kw.	830
55	harmful drinking.ti,ab,kw.	355
56	binge drinking.ti,ab,kw.	3839
57	alcohol-related.ti,ab,kw.	10,715
58	alcoholics.ti,ab,kw.	14,313
59	alcoholism.ti,ab,kw.	27,726
60	alcohol intoxication.ti,ab,kw.	2631
61	addict*.ti,ab,kw.	53,792
62	drug seeking.ti,ab,kw.	2296
63	polydrug.ti,ab,kw.	1348
64	bath salt*.ti,ab,kw.	308
65	cannabis.ti,ab,kw.	12,221
66	cocaine.ti,ab,kw.	34,890
67	coke.ti,ab,kw.	1657
68	crack.ti,ab,kw.	7033
69	designer drug*.ti,ab,kw.	1231
70	dextroamphetamine.ti,ab,kw.	640
71	ecstasy.ti,ab,kw.	3471
72	hallucinogen*.ti,ab,kw.	3180
73	hashish.ti,ab,kw.	559
74	heroin.ti,ab,kw.	12,723
75	hookah.ti,ab,kw.	495
76	legal high*.ti,ab,kw.	395
77	lsd.ti,ab,kw.	4631
78	lysergic acid.ti,ab,kw.	1889
79	marijuana.ti,ab,kw.	10,943
80	mdma.ti,ab,kw.	3715
81	meth.ti,ab,kw.	7061
82	methamphetamine*.ti,ab,kw.	9984
83	methylenedioxyamphetamine.ti,ab,kw.	569
84	opiate*.ti,ab,kw.	23,806
85	opioid*.ti,ab,kw.	71,719
86	opium.ti,ab,kw.	2207
87	phencyclidine*.ti,ab,kw.	4369
88	psychedelic*.ti,ab,kw.	628
89	psychoactive.ti,ab,kw.	8018
90	psychostimulant*.ti,ab,kw.	5544
91	street drug*.ti,ab,kw.	582
92	volatile solvent*.ti,ab,kw.	356
93	water pipe*.ti,ab,kw.	656
94	((alprazolam or amobarb* or amphetamine* or analgesic* or anthramycin or anxiolytic* or barbiturate* or benzodiazepine* or bromazepam or buprenorphine or chlordiazepoxide or clonazepam or clorazepate or cannabinoid* or codeine or demerol or devazepide or diazepam or dilaudid or dronabinol or duloxetine or endocannabinoid* or ephedrine or estazolam or estradiol or fentanyl or flumazenil or flunitrazepam or flurazepam or gabapentin or haloperidol or hydrocodone or hydromorphone or hypnotics or ketamine* or lorazepam or medazepam or meperidine or methadone or methylphenidate or midazolam or morphine or narcotic* or nitrazepam or oxazepam or oxycodone or oxycontin* or pentobarb* or pentobarbital or percocet or phenobarbital or piperazine* or pirenzepine or prazepam or pregabalin or propranolol or relaxant* or ritalin or secobarb* or sedative* or sleeping pill* or stimulant* or temazepam or tetrahydrocannabinol or tramadol or tramadol or tranquilizer* or triazolam or valium or vicodin or zolpidem) adj2 (abstinence or abstain or abuse or craving* or dependenc* or illegal or illicit or misuse or nonmedical or non-medical or nonprescription or non-prescription or overdose or prevent* or recovery or recreational or use* or withdrawal)).ti,ab,kw.	38,251
95	or/21-94 [ substance abuse terms ]	591,486
96	20 and 95	15,766
97	96 not (exp animals/ not (exp animals/ and humans/)) [remove animal studies]	14,357
98	limit 97 to (english language and yr=“1975 -Current”)	12,860

## Appendix 2

### Grey literature search strategy

#### Search engines

##### Strategy 1

- Google
- Google Scholar

** Note: prior to commencing search, log out of any google services (eg gmail) AND use browser’s “incognito” mode to ensure depersonalised results are shown.

****Note 2: copy and paste the entire search query into google and google scholar *exactly as written*. Do not add extra spaces, etc.

Two search queries, both to be used in both google AND google scholar:

**Table:**

1	substance|alcohol|drug|opioidabuse|misuse|withdrawal|abstinence|addict|craving|dependency|illegal|illicit|overdose|prevention|use|user|recreational|recovery exercise|"physical activity"|fitness|sedentary
2	substance|alcohol|drug|opioid abuse|misuse|withdrawal|abstinence|addict|craving|dependency|illegal|illicit|overdose|prevention|use|user|recreational|recovery exercise|"physical activity"|fitness|sedentary UK|"United Kingdom"|England|Britain|NHS

#### Grey lit databases

##### Strategy 1:

- Scottish Addiction Studies online library
- HRB National Drugs Library (use “all fields” search)
- NIDA International Drug Abuse Research Abstract Database (press enter to search; press enter with blank search to see total contents of the database)

Searches for the above databases:

****Note: Each of these searches use “physical activity” related terminology because the databases above are drug and alcohol related. Feel free to search for other relevant terminology as needed and record results in Excel.

**Table:**

1	exercise
2	"physical activity"
3	fitness
4	sport
5	sedentary

##### Strategy 2:

- British Library EThOS
- Tufts CEA Registry (Basic search --> Search for “articles”)

Searches for the above databases:

**Note: Each of these searches use a “physical activity” related term combined with a drug/alcohol misuse term. Feel free to search for other relevant combinations as needed and record results in Excel.

**Table:**

1	drugs AND exercise
2	opioids AND exercise
3	substance AND exercise
4	alcohol AND exercise
5	drugs AND "physical activity"
6	opioids AND "physical activity"
7	substance AND "physical activity"
8	alcohol AND "physical activity"

##### Strategy 3:

- NHS Evidence

Searches for the above database:

Note: Searches are split into two because of character limits of NHS Evidence search box

**Table:**

1	(substance OR alcohol OR drug OR opioid) AND (abuse OR misuse OR withdrawal OR abstinence OR addict OR craving OR dependency OR illegal OR illicit OR overdose OR prevention OR user OR recreational OR recovery) AND (exercise OR "physical activity") Select: "primary research" in sidebar
2	(substance OR alcohol OR drug OR opioid) AND (abuse OR misuse OR withdrawal OR abstinence OR addict OR craving OR dependency OR illegal OR illicit OR overdose OR prevention OR user OR recreational OR recovery) AND (fitness OR sedentary) Select: "primary research" in sidebar

##### Strategy 4:

- Open Grey

Search for the above database:

**Table:**

1	discipline:(05* OR 06*) lang:"en" (substance OR alcohol OR drug OR opioid) AND (abuse OR misuse OR withdrawal OR abstinence OR addict OR craving OR dependency OR illegal OR illicit OR overdose OR prevention OR use OR user OR recreational OR recovery) AND (exercise OR "physical activity" OR fitness OR sedentary)

##### Strategy 5:

- Database of promoting health effectiveness reviews (DoPHER) (Select “free text” radio button)

Search for the above database:

**Table:**

1	(substance OR alcohol OR drug OR opioid) AND (abuse OR misuse OR withdrawal OR abstinence OR addict OR craving OR dependency OR illegal OR illicit OR overdose OR prevention OR use OR user OR recreational OR recovery) AND (exercise OR "physical activity" OR fitness OR sedentary)

##### Strategy 6:

- Big Lottery Fund Database https://www.biglotteryfund.org.uk/funding/search-past-grants?page=0

Search for the above database:

**Table:**

1	Download all items from the database and create smart group in EndNote (*n* = 216,850) Any field ➔ word begins with ➔ substance*; alcohol*; drug*; opioid*; addict* AND Any field ➔ word begins with ➔ exercise*; fitness; "physical activity"; fitness; sedentary; sport* = 263 hits

##### Strategy 7:

To be conducted by AW.

- Proquest Dissertations and Theses

Using modified search strategies from the traditional database searches.

#### Grey lit searching procedures

##### Part 1: Database/ search engines

1. Conduct each search in each database.
2. For each search, record:
  - Date
  - Database and search query (in full)
  - Total number of hits (if available), or estimate if not.
  - Number of potentially relevant citations from search (not full websites, just papers/articles/etc.)
3. For each, screen first 100 hits (by title and abstract). If above 10% are relevant, screen next 100 hits, and so forth.
4. For each potentially relevant hit:
  - If the hit is an article or paper, add to reference management system. Ensure at a minimum that title and URL are captured.
  - If the hit is a website, add to excel spreadsheet for follow-up later (see below).
5. From all potentially relevant hits downloaded to reference management software, full-text screening of grey literature to be conducted by two people (half of results to be screened by each, with results checked by second person).

##### Part 2: Websites

1. For each website identified as potentially relevant (by experts, google searches, or any other sources), list website in Excel spreadsheet and conduct a search for relevant articles/papers.
2. For each website, search strategies might include any of the below, as necessary:
  - Handsearching of menus
  - Use of search bars with relevant terminology from searches above
  - Use of Google to search within the website, such as:
    i. “substance abuse” exercise *site:**website.com*
  - Use of Google to search within the website to find PDFs, such as:
    i. “substance abuse” exercise *site:**website.com* *filetype:pdf*
3. For each search, record in Excel:
  - Date
  - Website, including URL
  - Process used (e.g. search bar, hand searching, which menus used, targeted google site search, etc.)
  - Number of potentially relevant hits from each website.
4. For each hit, add to reference management system (it may be worthwhile to use a browser plug-in, such as from Zotero or Mendeley to semi-automate the process).
5. From all potentially relevant hits downloaded to reference management software, full text screening of grey literature to be conducted by two people (half of results to be screened by each, with results checked by second person).

## Additional file

Additional file 1: PRISMA-P 2015 Checklist. (DOCX 32 kb)

## Abbreviations

AMED: Allied and complementary medicine database
AUD: Alcohol use disorder
CHEERS: Consolidated Health Economics Evaluation Reporting Standards
CI: Confidence interval
CINAHL: The cumulative index to nursing and allied health literature
DOH: Department of Health
DSM-V: Diagnostic and statistical manual of mental health disorders, fifth edition
GRADE: Grading of recommendations, assessment, development, and evaluation
ICC: Intra-cluster correlation coefficient
MD: Mean differences
NHS: National Health Service
NIHR: National Institute of Health Research
PA: Physical activity
PPI: Patient and public involvement
PRISMA-P: Preferred Reporting Items for Systematic Reviews and Meta-analysis Protocols
RCT: Randomised controlled trial
ROBINS-I: Risk of bias in non-randomised studies of interventions
SMD: Standardised mean difference
SUD: Substance use disorder
UK: United Kingdom
